# Associations of Sleep Disorders With Depressive Symptoms in Early and Prodromal Parkinson’s Disease

**DOI:** 10.3389/fnagi.2022.898149

**Published:** 2022-06-10

**Authors:** Jiangnan Ma, Kaixin Dou, Ruize Liu, Yajin Liao, Zengqiang Yuan, Anmu Xie

**Affiliations:** ^1^Departmentof Neurology, Affiliated Hospital of Qingdao University, Qingdao, China; ^2^Department of Intensive Care Unit, Affiliated Qingdao Municipal Hospital of Qingdao University, Qingdao, China; ^3^The Brain Science Center, Beijing Institute of Basic Medical Sciences, Beijing, China

**Keywords:** Parkinson’s disease, sleep disorders, depression, rapid-eye-movement sleep behavior disorder, daytime sleepiness, autonomic dysfunction

## Abstract

**Background:**

Non-motor symptoms, including sleep disorders and depression, are common in Parkinson’s disease (PD). The purpose of our study is to explore the effect of sleep disorders, including the probable rapid eye movement (REM) sleep behavior disorder (pRBD) and the daytime sleepiness, on depressive symptoms in patients with early and prodromal PD.

**Methods:**

A total of 683 participants who obtained from the Parkinson Progression Markers Initiative (PPMI) were included, consisting of 423 individuals with early PD, 64 individuals with prodromal PD, and 196 healthy controls (HCs), who were followed up to 5 years from baseline. Multiple linear regression models and linear mixed-effects models were conducted to explore the relationship between sleep disorders and depression at baseline and longitudinally, respectively. Multiple linear regression models were used to further investigate the association between the change rates of daytime sleepiness score and depression-related score. Mediation analyses were also performed.

**Results:**

At baseline analysis, individuals with early and prodromal PD, who had higher RBD screening questionnaire (RBDSQ) score, or who were considered as pRBD, or who manifested specific behaviors of RBD (things falling down when sleep or disturbance of sleep), showed significantly the higher score of depression-related questionnaires. Our 5-year follow-up study showed that sleep disorders, including pRBD and daytime sleepiness, were associated with the increased depressive-related score in individuals with early and prodromal PD. Interestingly, we also found that the increased possibilities of daytime sleepiness were associated with depressive-related score. Finally, mediation analysis demonstrated that the relationship between RBD and depressive symptoms was partially mediated by autonomic symptoms, such as postural hypertension, salivation, dysphagia, and constipation.

**Conclusion:**

Our study shows that sleep disorders, including pRBD and daytime sleepiness, are associated with depression at baseline and longitudinally, which is partially mediated by the autonomic dysfunction in early and prodromal PD, with an implication that sleep management is of great value for disease surveillance.

## Introduction

Parkinson’s disease (PD) is a progressive neurodegenerative disease characterized by bradykinesia, tremor at rest, rigidity, and postural instability. However, various non-motor symptoms, including depression, sleep disorders, constipation, and hyposmia, often predate the motor symptoms, with the most common being depression (Postuma et al., 2015b; Schapira et al., 2017). In total, 90.3% of patients with PD had prodromal non-motor symptoms before the clinical diagnosis of PD (Durcan et al., 2019). Most notably rapid eye movement (REM), sleep behavior disorder (RBD), and olfactory, autonomic, neuropsychiatric, and cognitive dysfunctions, are relatively frequent in patients who progress into PD in the future (Moscovich et al., 2020).

Sleep disorders are considered one of the potential risks and progression factors of PD and a significant source of disability (Dauvilliers et al., 2018; Bohnen and Hu, 2019). Insomnia, RBD, excessive daytime sleepiness (EDS), and restless legs are common sleep disorders in PD. RBD, which is considered as parasomnia, is characterized by loss of normal muscle atonia, accompanied by violent motor manifestations of undesirable dreams during REM sleep (Dauvilliers et al., 2018). RBD is a promising risk marker of synucleinopathies such as PD. Approximately 50% patients with PD have RBD (Barone and Henchcliffe, 2018). Prodromal RBD is related to more rapid motor progression and non-motor PD subtypes, especially depressive disorders and cognitive decline (Ferini-Strambi et al., 2014; Barone and Henchcliffe, 2018; Bohnen and Hu, 2019). Comorbidity of idiopathic RBD and depression accelerates the neurodegenerative process (Ghazi Sherbaf et al., 2018). EDS is characterized by the inability to maintain wakefulness during the daytime, with sleep occurring unintentionally or at inappropriate times (Hustad and Aasly, 2020). EDS, as one of the earliest and most common non-motor symptoms of PD, substantially impacts the quality of life (Yousaf et al., 2018; Xiang et al., 2019). Patients who are tended by night shift nurses have a higher risk of PD, indicating that circadian rhythm disorder is related to the progression of PD (Zverev and Misiri, 2009; Hustad and Aasly, 2020). One of the pathologic features of PD is the aggregation of α-synuclein (α-syn) into proteinaceous inclusions (Lewy bodies, LBs). The spread of α-syn in the brain leads to the decline of the total cerebrospinal fluid (CSF) α-syn levels (Dolatshahi et al., 2018). Both pRBD and daytime sleepiness were significantly related to lower and decreased levels of CSF α-syn, suggesting a higher risk of PD progression (Wang et al., 2021).

The most common psychiatric symptom in PD is anxiety and depression (Grover et al., 2015). Compared with motor symptoms, sleep disorders, constipation, and other non-motor symptoms, depression has not been paid high attention. Meta-analyses have confirmed the incidence of depression in patients with PD is around 23% (Goodarzi et al., 2016), which is higher than in the ordinary elderly or in patients suffering from other chronic and disabling diseases (Chwastiak et al., 2002; Nilsson et al., 2002). The occurrence of depression is closely related to the course of PD. As the duration of PD progresses, the incidence of depression will increase exponentially (Cooney and Stacy, 2016). However, depression often overlaps with other symptoms of PD. Patients with PD and their caregivers are rarely aware of the manifestation of depression, which results in the delay in diagnosis and earlier treatment, and finally leads to the poor living quality of patients with PD. Many factors can predict the prognosis of PD, including age, depression, and gait disorder (Louis et al., 1999; Soh et al., 2011; Postuma and Berg, 2019; Sklerov et al., 2020). With the progress of the disease, depression will eventually affect the daily life activities in patients with PD (Ravina et al., 2007; Lawrence et al., 2014). Previous studies revealed that sleep disorders were independently associated with depressed mood and autonomic symptoms (Chung et al., 2013). PD and sleep disorders comorbidities indicate more non-motor symptoms, including depressive symptoms, lower quality of life, and poor cognition, and fatigue (Neikrug et al., 2013). Therefore, the early identification of high-risk patients for depressive symptoms is of great significance for improving the prognosis and the quality of life of patients with PD.

However, the associations of RBD and daytime sleepiness with depressive symptoms have not yet been clarified. Therefore, we examined the associations of sleep disorders with depressive symptoms in patients from the Parkinson’s Progression Markers Initiative (PPMI) database, by using cross-sectional and longitudinal analyses. In addition, mediating effect analyses were also performed for this study.

## Materials and Methods

### Participants From Parkinson Progression Markers Initiative

Data used in this study were obtained from the PPMI database, a comprehensive observational, international, multicenter study aimed at identifying PD progression biomarkers. The detailed information is available online,^1^ including inclusion/exclusion criteria, sites, complete lists of evaluations, and procedures. Our cohort was made up of healthy controls (HCs), prodromal PD (refers to the stage in which the condition has not yet progressed sufficiently to be defined as clinical PD, but several non-motor symptoms or signs of neurodegeneration are detectible), and early PD (refers to the stage where individuals were diagnosed with PD within 2 years but had not been treated with anti-parkinsonian therapy) (Postuma and Berg, 2019). In the PPMI database, hyposmia and RBD are used as the criteria for prodromal PD enrollment. Each individual participating in the cross-sectional analysis has completed the RBD screening questionnaire (RBDSQ) and Epworth sleep scale (ESS), and depression-related assessments (Geriatric Depression Scale [GDS]-short form and the Movement Disorder Society Unified Parkinson’s Disease Rating Scale [MDS-UPDRS] Part I Depressed Mood) at baseline. The clinical parameters included in our study were age, gender, years of education, blinded site, ethnicity, race, and Montreal Cognitive Assessment (MoCA) score. We did not find any participants with severe neurological disorders that may affect the results. All research procedures included in PPMI were approved by the local Institutional Review Boards of the participating centers. Written informed consent was obtained from all participants before study enrollment (Parkinson Progression, and Marker, 2011).

### Sleep Characterization in Parkinson Progression Markers Initiative

Sleep-related characteristics of participants were evaluated by RBDSQ and ESS, which were both patient self-assess questionnaires. RBDSQ consists of 10 items covering the clinical symptoms of RBD and dichotomic responses (Yes = 1 or No = 0) (Stiasny-Kolster et al., 2007). Items 1–4 enquire about the frequencies and contents of dreams and the relationship between dreams and actions. Item 5 enquires about self-injury and others’ injuries. Item 6 is divided into 4 subitems to assess specific conditions of abnormal movements, such as speaking in sleep, sudden limb movements, and complex movements. Items 7 and 8 enquire about the awakening at night, and item 9 enquires about the overall sleep. Item 10 concerning central nervous system diseases is excluded from our study. The total score of RBDSQ is 13 points. The best threshold for diagnosing RBD in the general population is 5 points. Probable RBD (pRBD) is screened using RBDSQ with a cutoff of 6 in patients with PD (Stiasny-Kolster et al., 2007; Miyamoto et al., 2009; Nomura et al., 2011). ESS is a handy questionnaire for patients to self-evaluate the degree of daytime sleepiness, which simulates eight scenarios to assess the possibility of drowsiness (never = 0, slight = 1, moderate = 2, and high = 3). The highest score of ESS is 24 points, and EDS is diagnosed with ESS ≥ 10 (Simuni et al., 2015; Wang et al., 2021).

### Depression Characterization in Parkinson Progression Markers Initiative

Depression-related characteristics of participants were evaluated by GDS (short form) and MDS-UPDRS Part I Depressed Mood. GDS comprises 15 items to evaluate the following symptoms of people over 56 years old: depression, decreased activity, irritability, withdrawal, and negative evaluation of the past and present. A higher score of GDS indicates a higher burden of depression. A score of 5 or more suggests depression. MDS-UPDRS is divided into five items. Item 1 concerning mentation, behavior, and mood includes the assessment of the degree of depressive symptoms (normal = 0, slight = 1, mild = 2, moderate = 3, and severe = 4). The higher the score, the more serious the depression is.

### Autonomic Dysfunction Assessments in Parkinson Progression Markers Initiative

Autonomic dysfunction has been confirmed to be related to depression and affects daily function in PD. The overall morbidity of autonomic dysfunction varies from 2% for urinary incontinence to 72% for constipation in patients with PD. To some extent, they were associated with disease duration, severity, or use of antiparkinsonian drugs (Visser et al., 2004). Autonomic dysfunctions were assessed by the Scales for Outcomes in Parkinson’s Disease-Autonomic (SCOPA-AUT), which consists of 25 items evaluating the six regions: gastrointestinal (GI, 7 subitems), urinary (6 subitems), cardiovascular (3 subitems), thermoregulatory (4 subitems), pupillomotor (1 subitem), and sexual (2 subitems for men and 2 subitems for women) dysfunction (never = 1, sometimes = 2, regularly = 4, and often = 4). A higher score of SCOPA-AUT indicates a higher burden of autonomic dysfunction (Sklerov et al., 2020).

### Data Analysis

Descriptive statistics were used to summarize the basic characteristics of participants. Age, gender, years of education, MoCA score, ethnicity, and race were considered as covariates. The differences between the three groups were compared by the Kruskal–Wallis test.

In the cross-sectional analyses, multiple linear regression models were used separately for combinations of every sleep subitems with the depression-related questionnaires, using the score of GDS or MDS-UPDRS Part I Depressed Mood as the dependent variable and sleep subitem as the independent variable after controlling possible covariates.

**FIGURE 1:**
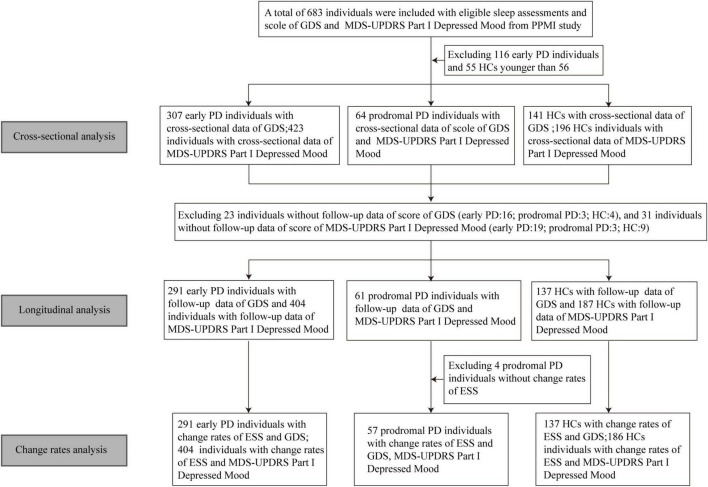
Flowchart of data analysis. GDS, geriatric depression scale; MDS-UPDRS, movement disorder society-sponsored revision unified Parkinson’s disease rating scale; PPMI, Parkinson’s progression markers initiative; PD, Parkinson’s disease; HC, healthy control; ESS, Epworth sleep scale.

In the longitudinal analyses, the impacts of baseline sleep behaviors on the longitudinal depression-related questionnaires were investigated by the fitted linear mixed-effects model. The interaction between time and baseline score of RBDSQ or ESS was used as a predictor. We additionally calculated change rates of the possibility of daytime sleepiness and the score of GDS or MDS-UPDRS Part I Depressed Mood during the follow-up by the sim function in the “arm” package with 1,000 replicates *via* linear mixed-effects models. Then multiple linear regression models were used to investigate the associations between the change rates of ESS subitems and depression-related questionnaires. Due to fewer patients with prodromal PD included, we combined patients with early PD with patients with prodromal PD into a group, collectively referred to as the PD group. All the above analyses were carried out in PD and HC participants separately. Figure 1 shows the flowchart of this study.

Mediation analyses were performed to investigate whether autonomic dysfunction, as measured by the SCOPA-AUT, might mediate the association between sleep disorders, as measured by the RBDSQ and ESS, with depressive symptoms, as measured by the GDS and MDS-UPDRS Part I Depressed Mood. The detection of the mediation effect is carried out by bootstrapping using the “mediation” package in R language.

Statistical significance was determined at a two-tailed *p*-value < 0.05. All statistical analyses and figure design were carried out in R version 4.1.2.

## Results

### Characterization of Participants at Baseline

The demographic characteristics of participants is shown in Table 1. A total of 683 individuals were included, consisting of 423 individuals with early PD, 64 individuals with prodromal PD, and 196 HCs. Individuals with prodromal PD had a mean age of 68.93 (SD: 5.788) years, which was more significant than those of early PD and HC groups. There were no statistically significant differences in the sex ratio of the samples among the three groups. The mean education years of different groups were all 15 years roughly. The participants were all cognitively well with the mean MoCA scores above 25. Total score of RBDSQ and ESS of prodromal PD group were higher than those of early PD and HC groups. The total score of MDS-UPDRS Part I Depressed Mood was lower in the HC group than early PD and prodromal groups. After excluding the population younger than 56 years old (including 116 individuals with early PD and 55 HCs), a total of 512 individuals were included in the statistics of GDS, consisting of 307 individuals with early PD, 64 individuals with prodromal PD, and 141 HCs. The total score of GDS was lower in the HC group than those of the other two groups. The proportion of participants with depression (assessed by GDS or MDS-UPDRS) and pRBD, and EDS in each group is shown in Table 1. In addition, there was no significant difference in the proportion of depression between prodromal and early PD groups, assessed by either GDS (*p* = 0.841) or MDS-UPDRS (*p* = 0.6079). In term of the abnormalities of sleep pattern, the pRBD (*p* = 1.338e−11), but not EDS (*p* = 0.2335) was significantly different between these two groups.

**TABLE 1:** Baseline characteristics of participants of three groups.

Variables	Early PD	Prodromal PD	HC	*p*-values
	*n* = 423	*n* = 64	*n* = 196	
Age				
Mean ± SD	61.66 ± 9.706	68.93 ± 5.788	60.82 ± 11.23	**2.53E**−**08**
(min, max)	(33.50, 84.88)	(58.91, 82.53)	(30.62, 83.68)	
Sex (n, %)				0.1064
male	277 (65.48%)	50 (78.12%)	126 (64.29%)	
female	146 (34.52%)	14 (21.88%)	70 (35.71%)	
Educate years				0.1387
Mean ± SD	15.56 ± 2.968	14.67 ± 4.511	16.04 ± 2.891	
(min, max)	(5.00, 26.00)	(0.00, 23.00)	(8.00, 24.00)	
MOCA				
Mean ± SD	27.13 ± 2.318	26.22 ± 3.494	28.23 ± 1.106	**7.03E**−**09**
(min, max)	(17, 30)	(11, 30)	(26, 30)	
RBDSQ total scores				**<2.2e**−**16**
Mean ± SD	4.121 ± 2.690	7.297 ± 3.736	2.827 ± 2.258	
(min, max)	(0, 12)	(1, 13)	(0, 11)	
ESS total scores				**0.03226**
Mean ± SD	5.801 ± 3.460	7.094 ± 4.245	5.621 ± 3.425	
(min, max)	(0, 20)	(0, 20)	(0, 19)	
GDS				**9.42E**−**07**
Mean ± SD	2.251 ± 2.348	2.250 ± 2.323	1.418 ± 2.306	
(min, max)	(0, 14)	(0, 10)	(0, 15)	
MDS-UPDRS Part I Depressed Mood				**0.004643**
Mean ± SD	0.2766 ± 0.5435	0.2500 ± 0.5909	0.1487 ± 0.4580	
(min, max)	(0, 4)	(0, 3)	(0, 4)	
Depression (n, %)				
Assessed by GDS	41 (13.44%)	8 (14.29%)	11 (7.69%)	
Assessed by MDS-UPDRS	100 (23.64%)	12 (18.75%)	24 (12.31%)	
pRBD (n, %)	43 (67.19%)	108 (25.53%)	25 (12.76%)	
EDS (n, %)	14 (21.88%)	66 (15.60%)	24 (12.24%)	

*PD, Parkinson’s disease; HC, healthy control; SD, standard deviation; MoCA, Montreal Cognitive Assessment; RBDSQ, rapid eye movement sleep behavior disorder screening questionnaire; ESS Epworth sleepiness scale, GDS, Geriatric Depression Scale, MDS-UPDRS, Movement Disorder Society-Sponsored Revision Unified Parkinson’s disease rating scale, pRBD, probable RBD, EDS, excessive daytime sleepiness.*

*The bold font of P-values means P value < 0.05.*

### The Associations of Sleep Disorders With Depressive Symptoms in Cross–Sectional Analyses

**FIGURE 2:**
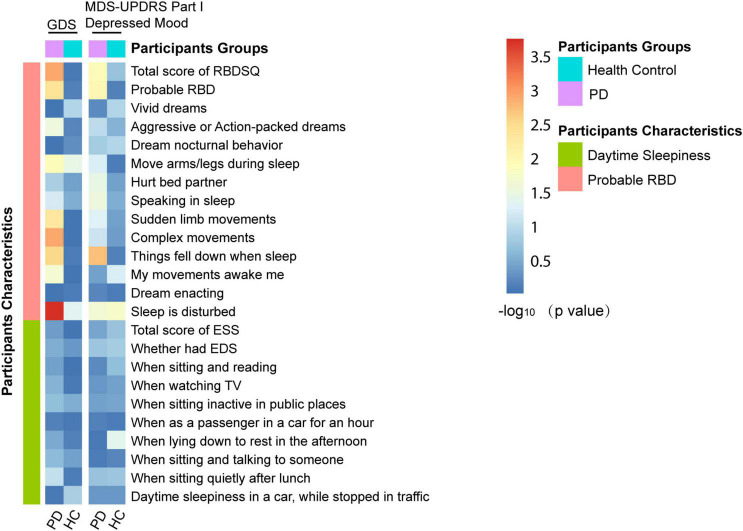
Associations of sleep disorders with depression in cross-sectional analyses.

**FIGURE 3:**
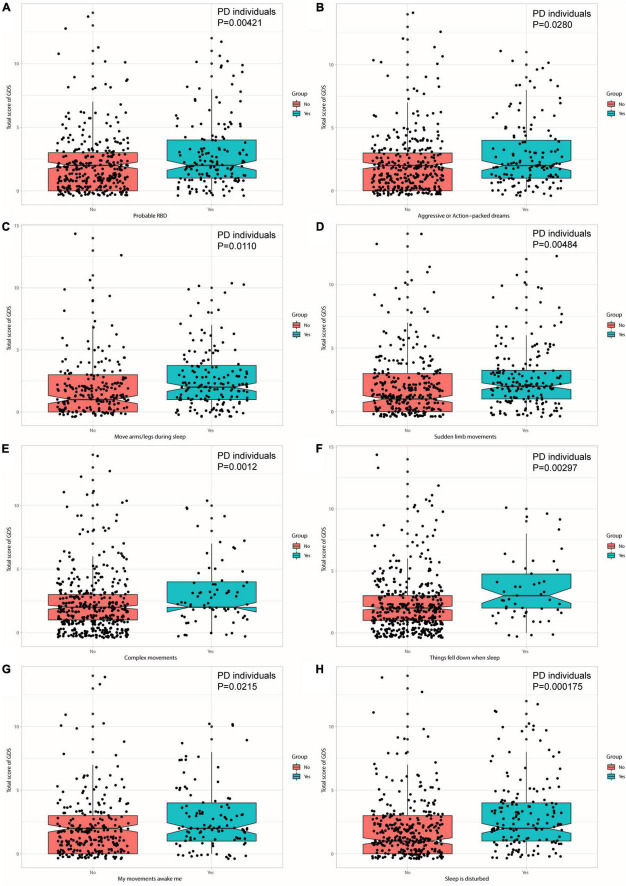
Individuals with PD with pRBD **(A)** and specific behaviors—aggressive or action-packed dreams **(B)**, moving arms/legs during sleep **(C)**, sudden limb movements **(D)**, complex movements **(E)**, things falling down when sleep **(F)**, being awakened by one’s own movements **(G)**, and disturbance of sleep **(H)** contribute to a higher score of GDS.

The associations between sleep disorders and depressive symptoms were revealed at baseline (Figure 2). Individuals with PD who had a higher RBDSQ score (β = 0.128939; *p* = 0.00121) or who were considered as pRBD (β = 0.771984; *p* = 0.00421) had a higher score of GDS. Moreover, individuals with PD who manifested specific behaviors of RBD, such as aggressive or action-packed dreams (β = 0.5929138; *p* = 0.0280), moving arms/legs during sleep (β = 0.656553; *p* = 0.0110), sudden limb movements (β = 0.739566; *p* = 0.00484), complex movements (β =−1.088929; *p* = 0.0012), things falling down when sleep (β =−1.189109; *p* = 0.00297), being awakened by one’s own movements (β = 0.636220; *p* = 0.0215), and disturbance of sleep (β = 1.002893; *p* = 0.000175) showed significantly a higher total score of GDS (Figure 3 and Supplementary Table 1). In addition, individuals with PD who had a higher RBDSQ score (β = 0.021491; *p* = 0.011292) or who were considered as pRBD (β = 0.143605; *p* = 0.009413) had a higher score of MDS-UPDRS Part I Depressed Mood. Moreover, individuals with PD who manifested specific behaviors of RBD, such as hurting bed partner (β = 0.139480; *p* = 0.035867), speaking in sleep (β = 0.114828; *p* = 0.03070), sudden limb movements (β = 0.106329; *p* = 0.048694), things falling down when sleep (β = 0.263490; *p* = 0.00185), and disturbance of sleep (β = 0.121387; *p* = 0.02337) showed significantly a higher total score of MDS-UPDRS Part I Depressed Mood (Supplementary Figure 1 and Supplementary Table 2). However, there was no significant association between daytime sleepiness and GDS or MDS-UPDRS Part I Depressed Mood in individuals with PD (Supplementary Tables 3, 4).

Furthermore, we found HCs who had specific behaviors of RBD, such as moving arms/legs during sleep (β = 0.90715; *p* = 0.0355) and disturbance of sleep (β = 0.89649; *p* = 0.0450) showed a higher total score of GDS, and who had disturbance of sleep (β = 0.1939777; *p* = 0.0179) showed a higher total score of MDS-UPDRS Part I Depressed Mood (Supplementary Tables 1, 2). There was no significant association between daytime sleepiness and GDS in HCs *via* cross-sectional analyses. And HCs with higher possibilities of daytime sleepiness when lying down to rest in the afternoon (β =−0.0689633; *p* = 0.0404) showed a significantly a higher total score of MDS-UPDRS Part I Depressed Mood (Supplementary Tables 3, 4).

### The Effects of Sleep Disorders at Baseline on Depressive Symptoms in Longitudinal Analyses

**FIGURE 4:**
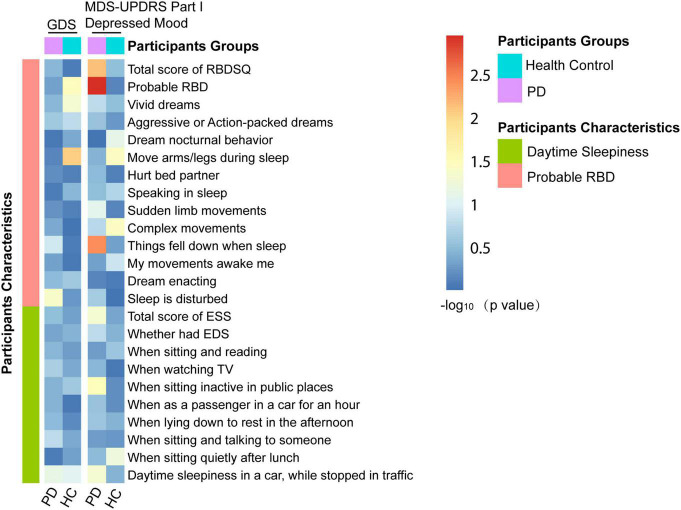
Associations of sleep disorders with depression in longitudinal analyses.

**FIGURE 5:**
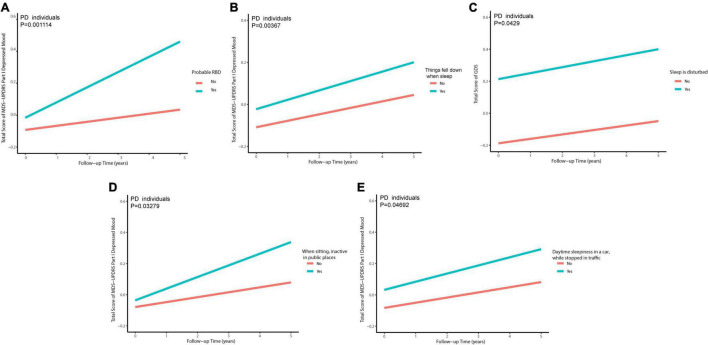
Individuals with PD with pRBD **(A)**, things falling down when sleep **(B)**, disturbance of sleep **(C)**, daytime sleepiness when sitting inactive in public places **(D)**, and daytime sleepiness in a car, while stopped in traffic **(E)** contribute to a higher score of depression-related scales in PD in longitudinal analyses.

Excluding 23 individuals without follow-up data of GDS (early PD: *n* = 16; prodromal PD: *n* = 3; HC: *n* = 4) and 31 individuals without follow-up data of MDS-UPDRS Part I Depressed Mood (early PD: *n* = 19; prodromal PD: *n* = 3; HC: *n* = 9) during the 5-year follow-up (baseline, 1st, 2nd, 3rd, 4th, and 5th annual follow-up): Associations of sleep disorders with depression during the follow-up are shown in Figure 4. As for individuals with PD with only disturbance of sleep (a specific behavior of RBD) were significantly associated with an increased total score of GDS (β = 0.1549074; *p* = 0.0429) during the follow-up (Supplementary Table 5). Individuals with PD who had a higher RBDSQ score (β = 2.872e−02; *p* = 0.00707), who were considered as pRBD (β = 2.201e−01; *p* = 0.001114), or had things falling down when sleep (β = 3.089e−01; *p* = 0.00367), showed an increasing trend of MDS-UPDRS Part I Depressed Mood (Supplementary Table 6). The PD participants with higher possibilities of daytime sleepiness when sitting inactive in public places (β = 9.717e−02; *p* = 0.03279) or stopped in traffic in a car (β = 1.830e−01; *p* = 0.04692) showed an increasing total score of MDS-UPDRS Part I Depressed Mood (Figure 5 and Supplementary Tables 7, 8).

In HCs, we did not identify any associations between daytime sleepiness and changes of depressive-related assessments. However, HCs who were considered as pRBD (β =−0.3860169; *p* = 0.0353) or had vivid dreams (β =−0.2347880; *p* = 0.0484) and moving arms/legs during sleep (β = 0.3253684; *p* = 0.0090) were found with an increased trend of GDS. Among the behaviors of RBD, only moving arms/legs during sleep (β = 0.228169; *p* = 0.035525) and complex movements (β = 0.472286; *p* = 0.036917) showed a significant development in MDS-UPDRS Part I Depressed Mood.

### Associations Between Longitudinal Changes in Scores of Epworth Sleep Scale and Change Rates of Geriatric Depression Scale or Movement Disorder Society Unified Parkinson’s Disease Rating Scale Part I Depressed Mood

Excluding 4 prodromal individuals without change rates of ESS: Associations between longitudinal change rates of daytime sleepiness and GDS or MDS-UPDRS Part I Depressed Mood in individuals with PD and HCs are shown in Supplementary Figure 2. As for individuals with PD, increased total score of ESS (β = 0.0937800; *p* = 0.001248) and increased possibilities of daytime sleepiness on four occasions (sitting and reading [β = 0.420573; *p* = 0.040591], sitting inactive in public places [β = 0.5232037; *p* = 0.003995], sitting quietly after lunch [β = 1.0065711; *p* = 4.95e−06], and stopped in traffic in a car [β = 0.3770992; *p* = 0.038161]) showed significant associations with a greater incline in GDS (Supplementary Table 9). While a greater increase in total score of ESS (β = 1.236e−02; *p* = 0.000279) and possibilities of daytime sleepiness on six occasions (sitting and reading [β = 5.877e−02; *p* = 0.0155], sitting inactive in public places [β = 6.492e−02; *p* = 0.00448], staying in a car as a passenger for an hour without a break [β = 7.551e−02; *p* = 0.00477], sitting and talking to someone [β = 6.852e−02; *p* = 0.00436], sitting quietly after lunch [β = 9.400e−02; *p* = 0.000386], and stopped in traffic in a car [β = 9.500e−02; *p* = 1.05e−05]) were significantly associated with a greater increase of MDS-UPDRS Part I Depressed Mood (Supplementary Figure 3 and Supplementary Table 10).

In HCs, change rates of the total score of ESS (β = 0.105651; *p* = 0.0191) and the possibility of daytime sleepiness only in a situation when sitting quietly after lunch (β = 1.015139; *p* = 0.000901) showed significant associations with change rates of GDS. Given that, an increasing daytime sleepiness score contributed to the greater incline in depressive-related scales, which was mainly found in individuals with PD.

### Analysis of Mediating Effect Between Sleep Disorders and Depressive Symptoms

**FIGURE 6:**
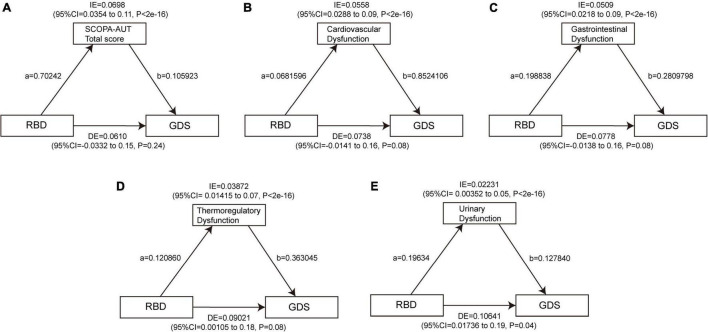
Autonomic dysfunctions mediate the effects of RBD on GDS. The significant mediating effect of RBD on GDS through the SCOPA-AUT total score **(A)**, cardiovascular dysfunction **(B)**, gastrointestinal dysfunction **(C)**, thermoregulatory dysfunction **(D)**, and urinary dysfunction **(E)** at baseline. Path a indicates the direct effect of RBD on autonomic symptoms. Path b indicates the direct effect of autonomic symptoms on depression. GDS, Geriatric Depression Scale; RBD, rapid eye movement sleep behavior disorder; SCOPA-AUT, Scales for Outcomes in Parkinson’s Disease-Autonomic.

**FIGURE 7:**
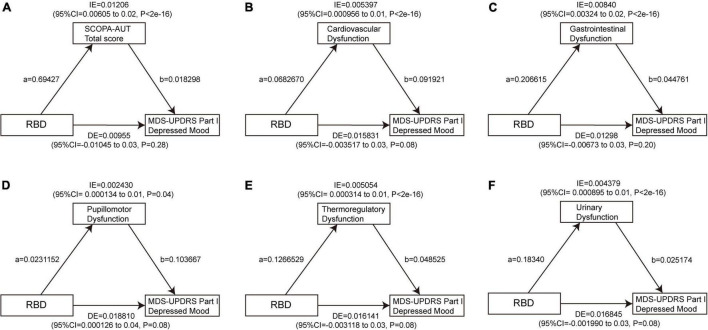
Autonomic dysfunctions mediate the impacts of RBD on MDS-UPDRS Part I Depressed Mood. The significant mediating effect of RBD on MDS-UPDRS Part I Depressed Mood through the SCOPA-AUT total score **(A)**, cardiovascular dysfunction **(B)**, gastrointestinal dysfunction **(C)**, pupillomotor dysfunction **(D)**, thermoregulatory dysfunction **(E)**, and urinary dysfunction **(F)** at baseline. Path a indicates the direct effect of RBD on autonomic symptoms. Path b indicates the direct effect of autonomic symptoms on depression. RBD, rapid eye movement sleep behavior disorder; MDS-UPDRS, movement disorder society unified Parkinson’s disease rating scale.

To date, there is no significant association found between daytime sleepiness and depression at baseline, so a mediation analysis was performed to investigate whether the association between RBD and depression is mediated by autonomic symptoms. The results showed a significant indirect effect of RBD on GDS through the SCOPA-AUT total score (β = 0.0698, 95% CI: 0.0354–0.11, *p* < 2e−16), cardiovascular subscore (β = 0.0558, 95% CI: 0.0288–0.09, *p* < 2e−16), GI subscore (β = 0.0509, 95% CI: 0.0218–0.09, *p* < 2e−16), thermoregulatory subscore (β = 0.03872, 95% CI: 0.01415–0.07, *p* < 2e−16), and urinary subscore (β = 0.02231, 95% CI: 0.00352–0.05, *p* < 2e−16) at baseline, indicating that autonomic dysfunction, especially cardiovascular, GI, and thermoregulatory dysfunction acted as full mediators between RBD and GDS, and also urinary dysfunction as a part mediator (Figure 6 and Supplementary Tables 11, 12). Similarly, the indirect effect of the total score of SCOPA-AUT (β = 0.01206, 95% CI: 0.00605–0.02, *p* < 2e−16), cardiovascular subscore (β = 0.005397, 95% CI: 0.000956–0.01, *p* < 2e−16), GI subscore (β = 0.00840, 95% CI: 0.00324–0.02, *p* < 2e−16), pupillomotor subscore (β = 0.00243, 95% CI: 0.000134–0.01, *p* = 0.04), thermoregulatory subscore (β = 0.005054, 95% CI: 0.000314–0.01, *p* < 2e−16), and urinary subscore (β = 0.004379, 95% CI: 0.000895–0.01, *p* < 2e−16) were strong predictors of increase in MDS-UPDRS Part I Depressed Mood at baseline (Figure 7 and Supplementary Tables 13, 14).

We further tested whether autonomic dysfunctions mediate the relationship between RBD and depression in HCs. The total score of SCOPA-AUT (β = 0.00976, 95% CI: 0.00102–0.02, *p* = 0.04) was a strong harbinger of the association between RBD and GDS. This analysis also revealed a significant indirect effect of RBD on MDS-UPDRS Part I Depressed Mood score through the SCOPA-AUT total score (β = 0.00976, 95% CI: 0.00102–0.02, *p* = 0.04), GI subscore (β = 0.0143, 95% CI: 0.0043–0.03, *p* < 2e−16), and sexual subscore (β = 0.00849, 95% CI: 0.00113–0.02, *p* = 0.04) at baseline.

Taken together, autonomic dysfunctions, especially cardiovascular, GI, thermoregulatory, and urinary dysfunction, are mediating effects between RBD and depressive symptoms in PD.

## Discussion

Non-motor symptoms in the early/prodromal stages of PD act as useful biomarkers for predicting the onset of motor symptoms and diagnosing PD, and identifying patients at risk of developing other complications (Bang et al., 2021).

Our findings suggested at baseline, a higher RBDSQ score was associated with a higher depression-related score in the PD group compared with HCs. In individuals with PD, patients with pRBD are significantly tend to concomitant with depression than patients with non-pRBD. In this regard, we further analyzed the correlation between each subitem of the RBDSQ and depression. For patients with geriatric PD over 56 years old, several specific behaviors of RBD, including subitem 2 (aggressive or action-packed dreams), subitem 4 (moving arms/legs during sleep), and subitems 6.2–6.4 (sudden limb movements, complex movements, and things falling down when sleep), and subitem 9 (disturbance of sleep), were significantly related to depressive symptoms, as measured by GDS. For patients with PD of all ages, subitem 5 (hurting bed partner), subitems 6.1, 6.2, and 6.4 (speaking in sleep, sudden limb movements, and things falling down when sleep), and subitem 9 (disturbance of sleep) showed significant positive correlations with depression, as measured by MDS-UPDRS Part I Depressed Mood. Accordingly, it has been reported that there is a close relationship between poor sleep quality in PD with depression (Rana et al., 2018). However, the causal relationship in the between remains to be further investigated.

In longitudinal analyses, for patients with geriatric PD over 56 years old, only subitem 9 (disturbance of sleep) of RBDSQ was a significant harbinger of depression, as measured by GDS. Individuals with PD with a higher RBDSQ score, pRBD or subitem 6.4 (things falling down when sleep) is of high possibility for depression incidence, basing on the scoring with MDS-UPDRS Part I Depressed Mood. For longitudinal analyses of daytime sleepiness with depression, we used the linear mixed-effects models and multiple linear regression models of change rates, respectively. Interestingly, we found that either the increased total score of ESS or the increased possibilities of daytime sleepiness on four occasions (sitting and reading, sitting inactive in public places, sitting quietly after lunch, and stopped in traffic in a car) are positively related with GDS scoring. Meantime, by using MDS-UPDRS Part I Depressed Mood, we observed that there is a significant correlation between the depression score with the high score of ESS, or the possibilities of daytime sleepiness on six occasions (sitting and reading, sitting inactive in public places, staying in a car as a passenger for an hour without a break, sitting and talking to someone, sitting quietly after lunch, and stopped in traffic in a car). Together, we argue that patients with PD with pRBD and daytime sleepiness are more prone to depression, based on the evaluation results with GDS or MDS-UPDRS. In consistent with the finding it has been found that there is a high frequency of sleep disorders during PD progression (Xu et al., 2021). Here, our work provides a detailed and longitudinal relationship of RBD and daytime sleepiness with depression, which might be used for the depression prediction in patients with the early stage and prodromal PD.

A multivariate analysis showed that older age, longer disease duration, and worse quality of sleep were independently associated with a higher SCOPA-AUT scale score (Arnao et al., 2015). It has been reported that the autonomic dysfunction is related to the development of depression of PD (Sagna et al., 2014). Idiopathic RBD is also significantly related to the mild-to-moderate autonomic dysfunction (Lee et al., 2015). We herein seek to investigate whether the autonomic dysfunction is involved depression development associated with the sleep disorders. By using the baseline mediating effect analyses, we found that RBD affected depression partially through autonomic dysfunction, especially the dysfunction of cardiovascular, GI, thermoregulatory, or urinary systems.

Postmortem autopsy of patients with PD with depression found a decrease in dopamine neurons and in the density of serotonin neurons in the dorsal and ventral tegmental areas of the raphe nuclei (Maillet et al., 2016; Rutten et al., 2017; Park et al., 2020). The biochemical basis of depression in PD may be related to extensive serotonin and reduced dopaminergic changes (Maillet et al., 2016; Patterson et al., 2019). We further analyze possible mechanisms at the anatomical and molecular levels. Increased α-syn oligomer in CSF and serum, and greater pathological density and range of synuclein could be observed in patients with PD with pRBD (Hu et al., 2015; Postuma et al., 2015a; Dušek et al., 2019). Areas involved in RBD are not only limited to the brainstem regions regulating REM sleep, but also may extend to other areas such as the olfactory system, the nigrostriatal system, and the autonomic system (Barone and Henchcliffe, 2018). Circadian rhythms affect the CSF production by regulating the accumulation and clearance of α-syn (Kudo et al., 2011; Benveniste et al., 2017; Sundaram et al., 2019). As a common symptom of circadian rhythm disruption, EDS manifests prior to Lewy pathology affects the topographic transmission of α-syn (Abbott et al., 2005, 2019). Therefore, α-syn is initially enriched in the lower brain stem, leading to sleep disturbances and autonomic dysfunction. In the later stage, the limbic system and neocortex are involved, followed by neuropsychiatric symptoms, such as depression (Braak et al., 2003, 2006; Beach et al., 2009; Park et al., 2020). It has been confirmed that RBD is associated with hyposmia, autonomic dysfunction, depression, cognitive impairment, and mild motor symptoms, which indicate diffuse α-syn pathology (Dušek et al., 2019). The direct effect of sleep disorders is fatigue (Chung et al., 2013; Stocchi et al., 2014), which is related to decreased serotonergic function in the basal ganglia and limbic structures (Remy et al., 2005; Pavese et al., 2010; Politis and Niccolini, 2015; Zuo et al., 2016). Depression in PD is attributable to serotonergic and noradrenergic lesions in the limbic system (Stocchi et al., 2014; Thobois et al., 2017; Jones et al., 2019). In addition, raphe serotonergic system is associated with sleep disorders and depression (Boileau et al., 2008; Politis et al., 2010; Xiao-Ling et al., 2020). Accumulation of phosphorylated α-syn depositing in the raphe nuclei contributes to depression (Xiao-Ling et al., 2020). Similarly, norepinephrine denervation is also involved in autonomic dysfunction in patients with PD, indicating that depression and autonomic dysfunction share a common neurochemical substrate (Sharabi and Goldstein, 2011). Damaged noradrenergic function in PD was also associated with RBD (Sommerauer et al., 2018; Pilotto et al., 2019). Taken together, depression in PD may be attributed to the disruption of neurotransmitter systems, such as dopamine (SN), serotonin (raphe nuclei), and noradrenaline (locus coeruleus) (Maillet et al., 2016; Jin et al., 2017; Hustad and Aasly, 2020; Park et al., 2020; Bang et al., 2021).

The above analyses prove that monitoring RBD and daytime sleepiness contributes to predicting and identifying depressive symptoms in patients with early PD and prodromal PD at baseline and within 5 years. Few previous studies have investigated this association, in particular the various subitems of RBD and daytime sleepiness. Previous studies reported that both depression and sleep disorders trigger a negative spiral in patients with PD, where one enhances the other. Sleep disturbances and depression severity share a bidirectional association (Kay et al., 2018; Sklerov et al., 2020). Therefore, independent clinical attention should be paid to these symptoms in patients with PD (Rutten et al., 2017; Kay et al., 2018).

However, our research still has some shortcomings. First of all, RBDSQ and ESS are both self-report questionnaires, which lack objective measures such as the polysomnography system for sleep monitoring. Second, the sample size is not large enough, with some participants losing follow-up, which may affect the reliability of results to a certain extent. The results require to be further verified by expanding of sample size and strengthening the follow-up.

## Conclusion

Sleep disorders were significantly associated with the higher and increased score of questionnaires assessing depression, suggesting a higher risk of progression to PD. Sleep disorders are identified as potential risk factors in predicting the depression and monitoring the progression of PD.

## Data Availability Statement

The datasets presented in this study can be found in online repositories. The names of the repository/repositories and accession number(s) can be found in the article/Supplementary Material.

## Ethics Statement

The studies involving human participants were reviewed and approved by the Local Institutional Review Boards of the participating centers. The patients/participants provided their written informed consent to participate in this study.

## Author Contributions

AX and ZY: conception and design of the study. JM, KD, and RL: acquisition and analysis of the data. JM, KD, and YL: drafting of the manuscript. All authors contributed to the article and approved the submitted version.

## Conflict of Interest

The authors declare that the research was conducted in the absence of any commercial or financial relationships that could be construed as a potential conflict of interest.

## Publisher’s Note

All claims expressed in this article are solely those of the authors and do not necessarily represent those of their affiliated organizations, or those of the publisher, the editors and the reviewers. Any product that may be evaluated in this article, or claim that may be made by its manufacturer, is not guaranteed or endorsed by the publisher.

## Footnotes

1
https://www.ppmi-info.org
