# Supplementary material for: Associations of Sleep Disorders With Depressive Symptoms in Early and Prodromal Parkinson’s Disease
Source: Front Aging Neurosci. 2022 Jun 10;14:898149. doi: 10.3389/fnagi.2022.898149 (PMC9226450; doi:10.3389/fnagi.2022.898149)
Supplement: Supplementary file 1 [file Data_Sheet_1.docx]

Supplementary Material


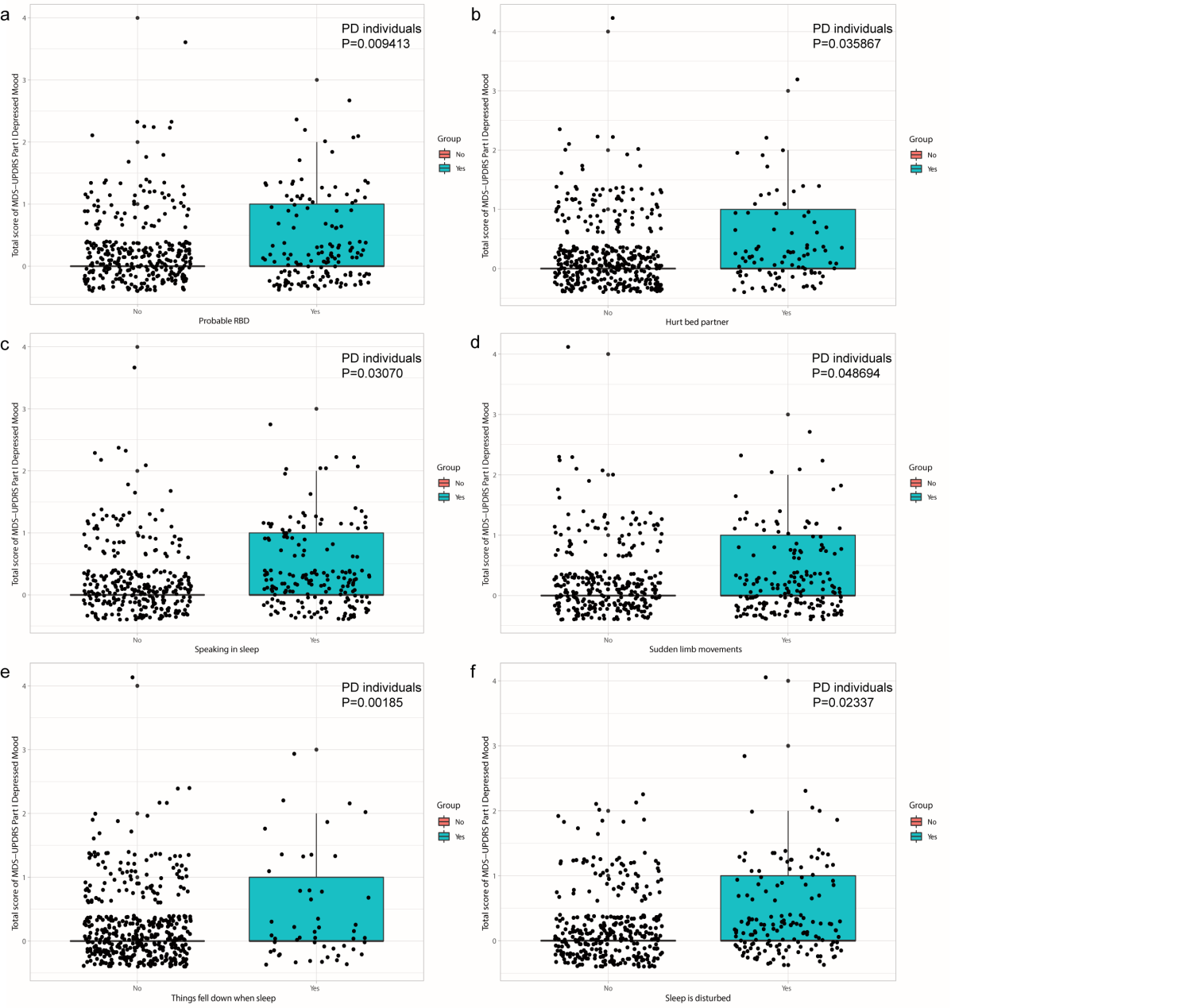


Supplementary Fig 1. PD individuals with pRBD (a) or specific behaviors -hurting bed partner (b), speaking in sleep (c), sudden limb movements (d), things falling down when sleep (e) and disturbance of sleep (f) contribute to higher score of MDS-UPDRS Part I Depressed Mood scale.


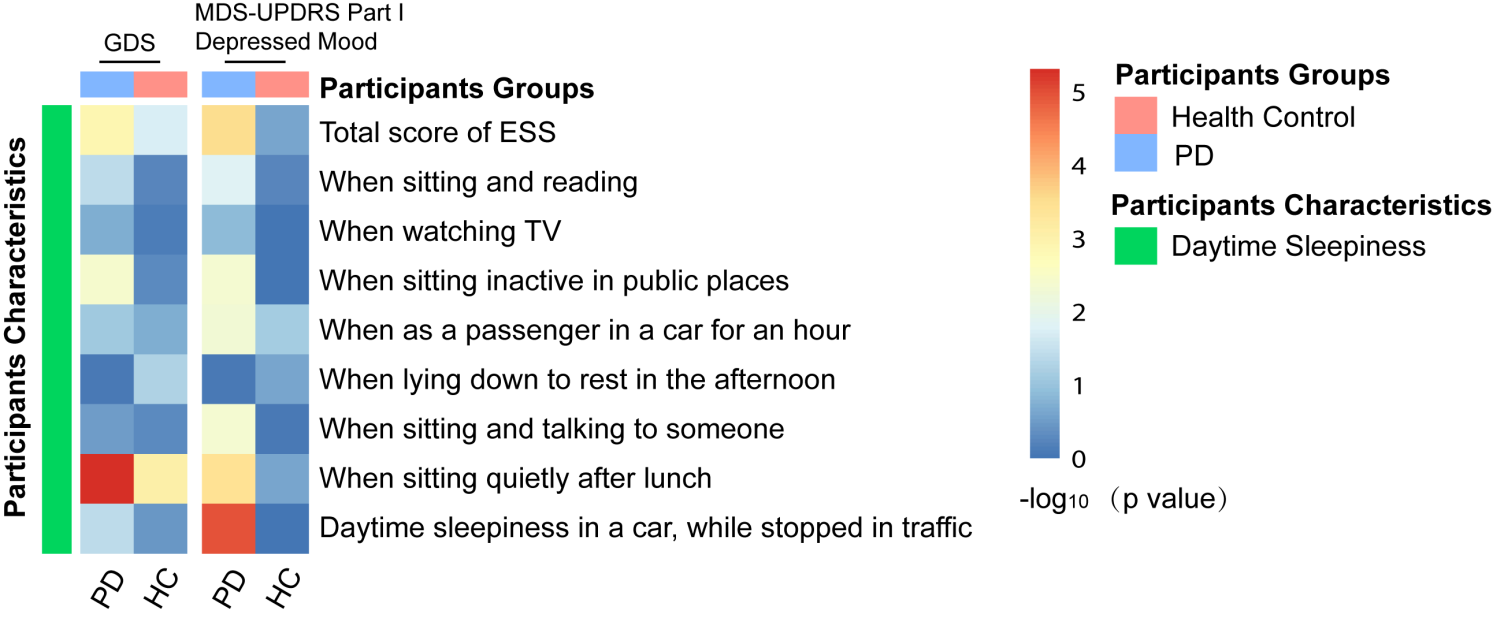
Supplementary Fig. 2 Associations between change rates of daytime sleepiness and changes of GDS and MDS-UPDRS Part I Depressed Mood in PD and HC individuals.


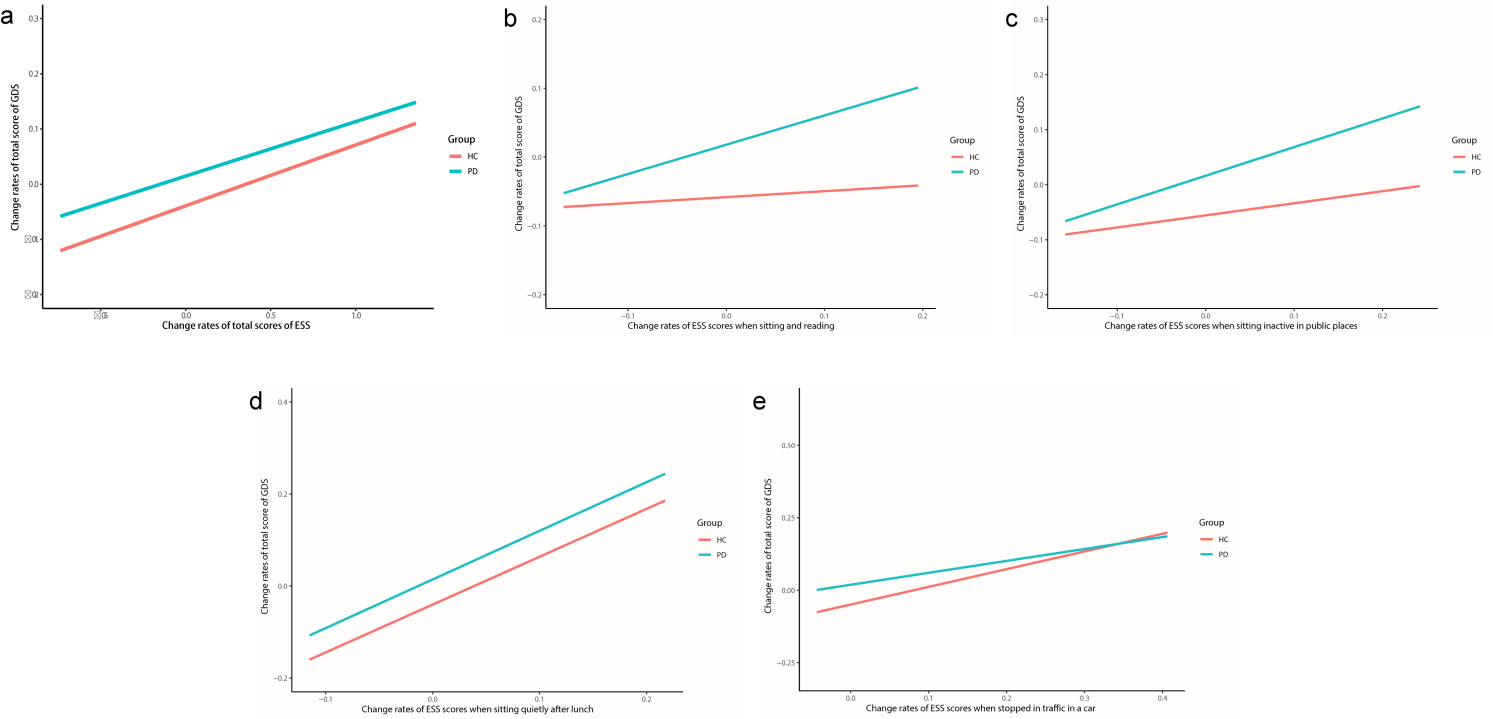


Supplementary Fig. 3 Associations between change rates of daytime sleepiness score and GDS in PD and HC individuals. Increased change rates of total scores of ESS (a), possibilities of daytime sleepiness when sitting and reading (b), when sitting inactive in public places (c), when sitting quietly after lunch (d) and when stopped in traffic in a car (e) in PD are associated with faster increase of change rates of score of GDS.


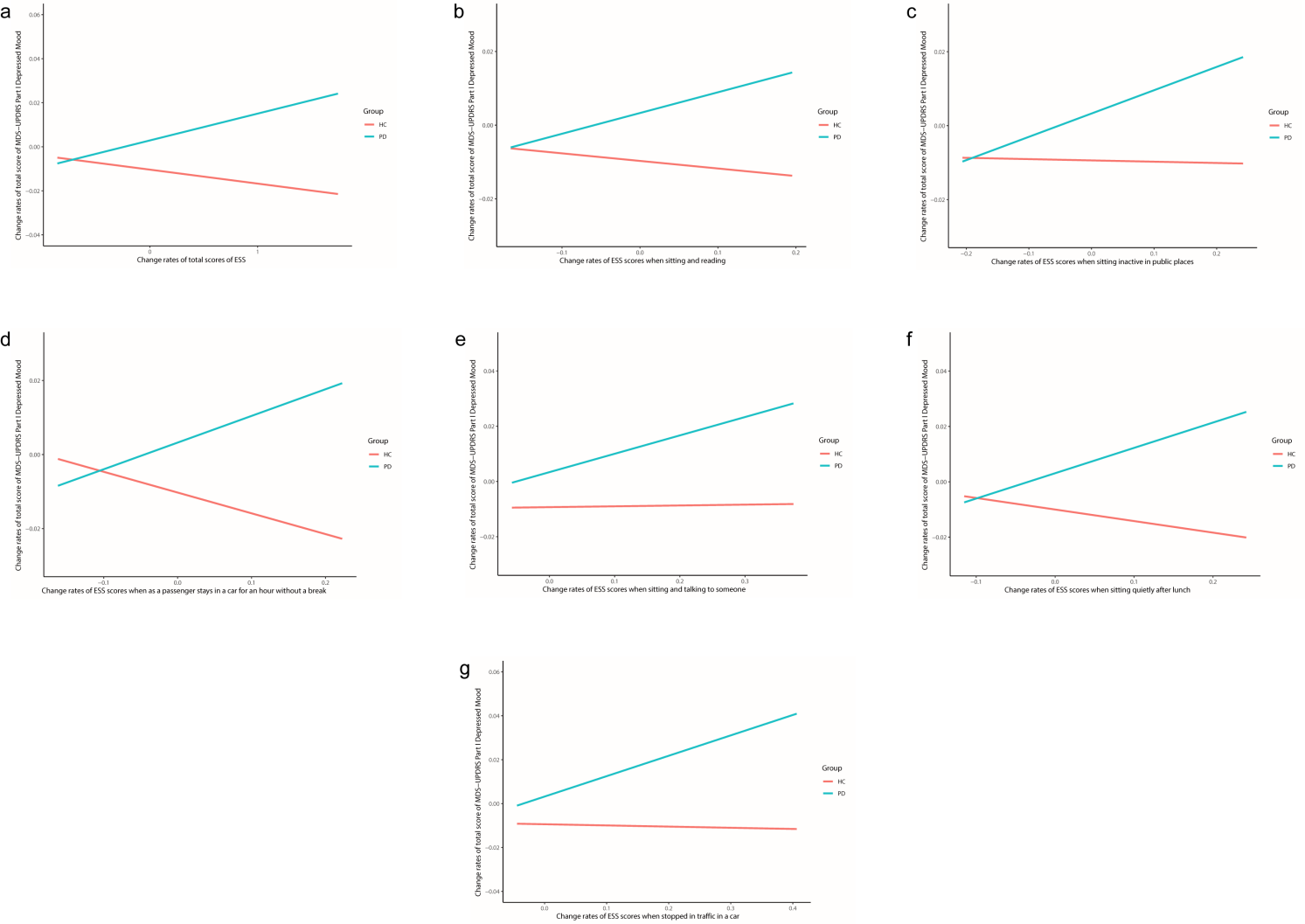


Supplementary Fig. 4 Associations between change rates of daytime sleepiness scores and change rates of MDS-UPDRS Part I Depressed Mood in PD and HC individuals. Increased change rates of total scores of ESS (a), possibilities of daytime sleepiness when sitting and reading (b), when sitting inactive in public places (c), when staying in a car as a passenger for an hour without a break (d), when sitting and talking to someone (e), when sitting quietly after lunch (f) and when stopped in traffic in a car (g) in PD are associated with faster increase of change rates of score of MDS-UPDRS Part I Depressed Mood.

| **Table 1. Associations of RBD with GDS in PD and HC individuals in cross-sectional study.** | | | | | |
| --- | --- | --- | --- | --- | --- |
| **Sleep characteristics** | **PD** | |  | **HC** | |
|  | **β** | **P** |  | **β** | **P** |
| Total score of RBDSQ | 0.128939 | **0.00121** |  | 0.01946 | 0.8212 |
| Probable RBD (total score of RBDSQ≥6) | 0.771984 | **0.00421** |  | -0.22280 | 0.6964 |
| Vivid Dreams | -0.034557 | 0.8915 |  | -0.62677 | 0.1171 |
| Aggressive or Action-packed dreams | 0.5929138 | **0.0280** |  | -0.22499 | 0.6263 |
| Dream nocturnal behavior | 0.030537 | 0.9181 |  | -0.30202 | 0.5507 |
| Move arms/legs during sleep | 0.656553 | **0.0110** |  | 0.90715 | **0.0355** |
| Hurt bed partner | 0.454859 | 0.139 |  | -0.79423 | 0.4024 |
| Speaking in sleep | 0.471305 | 0.0649 |  | -0.48053 | 0.2978 |
| Sudden limb movements | 0.739566 | **0.00484** |  | -0.04115 | 0.9382 |
| Complex movements | 1.088929 | **0.0012** |  | 0.13065 | 0.8996 |
| Things fell down when sleep | 1.189109 | **0.00297** |  | -0.29981 | 0.7555 |
| My movements awake me | 0.636220 | **0.0215** |  | 0.02620 | 0.9632 |
| Dream enacting | 0.013043 | 0.9604 |  | 0.12360 | 0.7522 |
| Sleep is disturbed | 1.002893 | **0.000175** |  | 0.89649 | **0.0450** |

RBD: Rapid eye movement sleep Behavior Disorder; GDS: Geriatric Depression Scale;PD: Parkinson’s disease;HC: Healthy controls; RBDSQ: RBD Screening Questionnaire

| **Table 2. Associations of RBD with** **MDS-UPDRS Part I Depressed Mood in PD and HC individuals in cross-sectional study.** | | | | | |
| --- | --- | --- | --- | --- | --- |
| **Sleep characteristics** | **PD** | |  | **HC** | |
|  | **β** | **P** |  | **β** | **P** |
| Total score of RBDSQ | 0.021491 | **0.011292** |  | 0.0192544 | 0.2038 |
| Probable RBD (total score of RBDSQ≥6) | 0.143605 | **0.009413** |  | 0.0375626 | 0.7119 |
| Vivid Dreams | 0.028190 | 0.58165 |  | -0.1127363 | 0.1128 |
| Aggressive or Action-packed dreams | 0.090772 | 0.09836 |  | -0.0900990 | 0.2767 |
| Dream nocturnal behavior | -0.085228 | 0.15414 |  | 0.1315865 | 0.1127 |
| Move arms/legs during sleep | 0.097888 | 0.059463 |  | -0.0239674 | 0.7536 |
| Hurt bed partner | 0.139480 | **0.035867** |  | -0.1429176 | 0.3931 |
| Speaking in sleep | 0.114828 | **0.03070** |  | 7.929e-02 | 0.3077 |
| Sudden limb movements | 0.106329 | **0.048694** |  | 0.0777031 | 0.3911 |
| Complex movements | 0.129666 | 0.07492 |  | 0.1403205 | 0.4079 |
| Things fell down when sleep | 0.263490 | **0.00185** |  | 5.778e-02 | 0.7350 |
| My movements awake me | 0.050089 | 0.37871 |  | 0.1845616 | 0.0565 |
| Dream enacting | -0.024906 | 0.63119 |  | -0.0218631 | 0.7524 |
| Sleep is disturbed | 0.121387 | **0.02337** |  | 0.1939777 | **0.0179** |

RBD: Rapid eye movement sleep Behavior Disorder; MDS-UPDRS: Movement Disorder Society Unified Parkinson’s Disease Rating Scale;PD: Parkinson’s disease;HC: Healthy controls; RBDSQ: RBD Screening Questionnaire

| **Table 3. Associations of daytime sleepiness with GDS in PD and HC individuals in cross-sectional study.** | | | | | |
| --- | --- | --- | --- | --- | --- |
| **Sleep characteristics** | **PD** | |  | **HC** | |
|  | **β** | **P** |  | **β** | **P** |
| Total score of ESS | 0.026395 | 0.4404 |  | -0.00848 | 0.8893 |
| Whether had EDS (total score of ESS ≥ 10) | 0.3472392 | 0.2991 |  | -0.45751 | 0.4824 |
| **Possibilities of daytime sleepiness** |  |  |  |  |  |
| Possibilities of daytime sleepiness when sitting and reading | 0.126991 | 0.3853 |  | -0.02396 | 0.9320 |
| Possibilities of daytime sleepiness when watching TV | 0.155464 | 0.283 |  | -0.05158 | 0.8443 |
| Possibilities of daytime sleepiness when sitting, inactive in a public place | 0.2477454 | 0.2045 |  | 0.31372 | 0.2956 |
| Possibilities of daytime sleepiness when one as a passenger stays in a car for an hour without a break | 0.053491 | 0.7029 |  | -0.05021 | 0.8280 |
| Possibilities of daytime sleepiness when lying down to rest in the afternoon | -0.126315 | 0.3187 |  | -0.06862 | 0.7219 |
| Possibilities of daytime sleepiness when sitting and talking to someone | -0.504899 | 0.2286 |  | -1.98537 | 0.3851 |
| Possibilities of daytime sleepiness when sitting quietly after lunch | 0.27252 | 0.0937 |  | 0.08702 | 0.7599 |
| Possibilities of daytime sleepiness in a car, while stopped in traffic | 0.082895 | 0.8475 |  | -0.87475 | 0.1336 |

GDS: Geriatric Depression Scale;PD: Parkinson’s disease;HC: Healthy controls; ESS: Epworth sleep scale;EDS: Excessive daytime sleepiness

| **Table 4. Associations of daytime sleepiness with** **MDS-UPDRS Part I Depressed Mood in PD and HC individuals in cross-sectional study.** | | | | | |
| --- | --- | --- | --- | --- | --- |
| **Sleep characteristics** | **PD** | |  | **HC** | |
|  | **β** | **P** |  | **β** | **P** |
| Total score of ESS | 0.006362 | 0.36691 |  | -1.287e-02 | 0.1960 |
| Whether had EDS (total score of ESS ≥ 10) | 0.089789 | 0.18322 |  | -0.1494522 | 0.1528 |
| **Possibilities of daytime sleepiness** |  |  |  |  |  |
| Possibilities of daytime sleepiness when sitting and reading | 0.015164 | 0.60898 |  | -0.0573562 | 0.2130 |
| Possibilities of daytime sleepiness when watching TV | 0.020182 | 0.48262 |  | 0.0384299 | 0.3761 |
| Possibilities of daytime sleepiness when sitting, inactive in a public place | 0.034442 | 0.37516 |  | -0.0456376 | 0.3583 |
| Possibilities of daytime sleepiness when one as a passenger stays in a car for an hour without a break | 0.010172 | 0.72586 |  | -0.0112957 | 0.7701 |
| Possibilities of daytime sleepiness when lying down to rest in the afternoon | 0.004227 | 0.86994 |  | -0.0689633 | **0.0404** |
| Possibilities of daytime sleepiness when sitting and talking to someone | -0.025525 | 0.7833 |  | -0.1173938 | 0.6652 |
| Possibilities of daytime sleepiness when sitting quietly after lunch | 0.042939 | 0.19145 |  | -0.0657345 | 0.1829 |
| Possibilities of daytime sleepiness in a car, while stopped in traffic | 0.064140 | 0.46976 |  | -0.0795799 | 0.4483 |

MDS-UPDRS: Movement Disorder Society Unified Parkinson’s Disease Rating Scale;PD: Parkinson’s disease;HC: Healthy controls; ESS: Epworth sleep scale;EDS: Excessive daytime sleepiness

| **Table 5. Associations of RBD with GDS in PD and HC individuals in longitudinal study.** | | | | | |
| --- | --- | --- | --- | --- | --- |
| **Sleep characteristics** | **PD** | |  | **HC** | |
|  | **β** | **P** |  | **β** | **P** |
| Total score of RBDSQ | 0.0117698 | 0.3530 |  | 0.0063929 | 0.8166 |
| Probable RBD (total score of RBDSQ≥6) | 0.0560510 | 0.4849 |  | -0.3860169 | **0.0353** |
| Vivid Dreams | -0.0685038 | 0.3398 |  | -0.2347880 | **0.0484** |
| Aggressive or Action-packed dreams | -0.0916846 | 0.2394 |  | -0.1875022 | 0.1652 |
| Dream nocturnal behavior | -0.0091083 | 0.9065 |  | -0.1116068 | 0.4201 |
| Move arms/legs during sleep | 0.0249105 | 0.7354 |  | 0.3253684 | **0.0090** |
| Hurt bed partner | -0.0451448 | 0.6327 |  | 0.0930113 | 0.7492 |
| Speaking in sleep | -0.0171014 | 0.8195 |  | -0.1296731 | 0.3518 |
| Sudden limb movements | 0.0402094 | 0.6019 |  | 0.0392262 | 0.7976 |
| Complex movements | 0.0816510 | 0.4172 |  | -0.0128707 | 0.9618 |
| Things fell down when sleep | 0.1850010 | 0.1217 |  | -0.0672939 | 0.8184 |
| My movements awake me | 0.0548246 | 0.4836 |  | -0.0195798 | 0.8978 |
| Dream enacting | -0.0715242 | 0.3315 |  | -0.1349651 | 0.2390 |
| Sleep is disturbed | 0.1549074 | **0.0429** |  | 0.1162931 | 0.5706 |

RBD: Rapid eye movement sleep Behavior Disorder; GDS: Geriatric Depression Scale;PD: Parkinson’s disease;HC: Healthy controls; RBDSQ: RBD Screening Questionnaire

| **Table 6. Associations of RBD with MDS-UPDRS Part I Depressed Mood in PD and HC individuals in longitudinal study.** | | | | | |
| --- | --- | --- | --- | --- | --- |
| **Sleep characteristics** | **PD** | |  | **HC** | |
|  | **β** | **P** |  | **β** | **P** |
| Total score of RBDSQ | 2.872e-02 | **0.00707** |  | 0.025200 | 0.299592 |
| Probable RBD (total score of RBDSQ≥6) | 2.201e-01 | **0.001114** |  | 0.054320 | 0.736732 |
| Vivid Dreams | 8.569e-02 | 0.16635 |  | -0.109820 | 0.308521 |
| Aggressive or Action-packed dreams | 7.311e-02 | 0.28029 |  | -0.071556 | 0.561049 |
| Dream nocturnal behavior | -1.550e-03 | 0.98240 |  | 0.193674 | 0.078568 |
| Move arms/legs during sleep | 5.809e-02 | 0.36106 |  | 0.228169 | **0.035525** |
| Hurt bed partner | 8.397e-02 | 0.323477 |  | -0.074994 | 0.756906 |
| Speaking in sleep | 6.688e-02 | 0.30602 |  | 1.552e-01 | 0.191681 |
| Sudden limb movements | 1.143e-01 | 0.08373 |  | 0.049353 | 0.709827 |
| Complex movements | 1.238e-01 | 0.16882 |  | 0.472286 | **0.036917** |
| Things fell down when sleep | 3.089e-01 | **0.00367** |  | -0.159889 | 0.494247 |
| My movements awake me | 4.686e-02 | 0.49854 |  | 0.494247 | 0.135059 |
| Dream enacting | 2.310e-02 | 0.71425 |  | -0.022558 | 0.82225 |
| Sleep is disturbed | 7.871e-02 | 0.230411 |  | 0.003923 | 0.973976 |

RBD: Rapid eye movement sleep Behavior Disorder; MDS-UPDRS: Movement Disorder Society Unified Parkinson’s Disease Rating Scale;PD: Parkinson’s disease;HC: Healthy controls; RBDSQ: RBD Screening Questionnaire

| **Table 7. Associations of daytime sleepiness with GDS in PD and HC individuals in longitudinal study.** | | | | | |
| --- | --- | --- | --- | --- | --- |
| **Sleep characteristics** | **PD** | |  | **HC** | |
|  | **β** | **P** |  | **β** | **P** |
| Total score of ESS | 0.0101777 | 0.3052 |  | 0.0134472 | 0.4838 |
| Whether had EDS (total score of ESS ≥ 10) | 0.0658944 | 0.4651 |  | -0.1656758 | 0.3676 |
| **Possibilities of daytime sleepiness** |  |  |  |  |  |
| Possibilities of daytime sleepiness when sitting and reading | -0.0411299 | 0.3322 |  | -0.0535424 | 0.5168 |
| Possibilities of daytime sleepiness when watching TV | 0.0523761 | 0.2160 |  | 0.0607712 | 0.4231 |
| Possibilities of daytime sleepiness when sitting, inactive in a public place | 0.0493378 | 0.3619 |  | 0.1078839 | 0.2419 |
| Possibilities of daytime sleepiness when one as a passenger stays in a car for an hour without a break | 0.0373394 | 0.3684 |  | 0.0085836 | 0.9060 |
| Possibilities of daytime sleepiness when lying down to rest in the afternoon | -0.0364636 | 0.3196 |  | 0.0245913 | 0.6700 |
| Possibilities of daytime sleepiness when sitting and talking to someone | 0.1308179 | 0.1575 |  | 0.2520147 | 0.4343 |
| Possibilities of daytime sleepiness when sitting quietly after lunch | 0.01061214 | 0.8093 |  | 0.0597853 | 0.4675 |
| Possibilities of daytime sleepiness in a car, while stopped in traffic | 0.2027354 | 0.0710 |  | -0.3203694 | 0.0886 |

GDS: Geriatric Depression Scale;PD: Parkinson’s disease;HC: Healthy controls; ESS: Epworth sleep scale;EDS: Excessive daytime sleepiness

| **Table 8. Associations of daytime sleepiness with MDS-UPDRS Part I Depressed Mood in PD and HC individuals in longitudinal study.** | | | | | |
| --- | --- | --- | --- | --- | --- |
| **Sleep characteristics** | **PD** | |  | **HC** | |
|  | **β** | **P** |  | **β** | **P** |
| Total score of ESS | 1.601e-02 | **0.04966** |  | -0.012456 | 0.444500 |
| Whether had EDS (total score of ESS ≥ 10) | 1.083e-01 | 0.16533 |  | -0.142498 | 0.35899 |
| **Possibilities of daytime sleepiness** |  |  |  |  |  |
| Possibilities of daytime sleepiness when sitting and reading | 2.330e-02 | 0.51195 |  | -0.078175 | 0.25914 |
| Possibilities of daytime sleepiness when watching TV | 3.391e-02 | 0.33904 |  | 0.011365 | 0.860718 |
| Possibilities of daytime sleepiness when sitting, inactive in public places | 9.717e-02 | **0.03279** |  | -0.036144 | 0.646080 |
| Possibilities of daytime sleepiness when one as a passenger stays in a car for an hour without a break | 3.833e-02 | 0.27131 |  | 0.030994 | 0.6221 |
| Possibilities of daytime sleepiness when lying down to rest in the afternoon | 3.470e-02 | 0.26416 |  | -0.047425 | 0.355549 |
| Possibilities of daytime sleepiness when sitting and talking to someone | 6.065e-02 | 0.498728 |  | -0.158008 | 0.540842 |
| Possibilities of daytime sleepiness when sitting quietly after lunch | 3.747e-02 | 0.32539 |  | -0.137722 | 0.057705 |
| Possibilities of daytime sleepiness in a car, while stopped in traffic | 1.830e-01 | **0.04692** |  | 1.450e-01 | 0.374209 |

MDS-UPDRS: Movement Disorder Society Unified Parkinson’s Disease Rating Scale;PD: Parkinson’s disease;HC: Healthy controls; ESS: Epworth sleep scale;EDS: Excessive daytime sleepiness

| **Table 9. Associations between change rates of daytime sleepiness and changes of GDS in PD and HC individuals.** | | | | | |
| --- | --- | --- | --- | --- | --- |
| **Sleep characteristics** | **PD** | |  | **HC** | |
|  | **β** | **P** |  | **β** | **P** |
| Total score of ESS | 0.0937800 | **0.001248** |  | 0.105651 | **0.0191** |
| Possibilities of daytime sleepiness when sitting and reading | 0.420573 | **0.040591** |  | 0.149379 | 0.577 |
| Possibilities of daytime sleepiness when watching TV | 0.2315843 | 0.19341 |  | 0.077951 | 0.758 |
| Possibilities of daytime sleepiness when sitting inactive in public places | 0.5232037 | **0.003995** |  | 0.187777 | 0.4942 |
| Possibilities of daytime sleepiness when one as a passenger stays in a car for an hour without a break | 0.4043891 | 0.076813 |  | 0.366809 | 0.193 |
| Possibilities of daytime sleepiness when lying down to rest in the afternoon | -0.0488508 | 0.821834 |  | 0.497425 | 0.0544 |
| Possibilities of daytime sleepiness when sitting and talking to someone | 0.1831542 | 0.316485 |  | 0.325757 | 0.509 |
| Possibilities of daytime sleepiness when sitting quietly after lunch | 1.0065711 | **4.95e-06** |  | 1.015139 | **0.000901** |
| Possibilities of daytime sleepiness in a car, while stopped in traffic | 0.3770992 | **0.038161** |  | 0.528551 | 0.375 |

GDS: Geriatric Depression Scale;PD: Parkinson’s disease;HC: Healthy controls; ESS: Epworth sleep scale

| **Table 10. Associations between change rates of daytime sleepiness and changes of MDS-UPDRS Part I Depressed Mood in PD and HC individuals** | | | | | |
| --- | --- | --- | --- | --- | --- |
| **Sleep characteristics** | **PD** | |  | **HC** | |
|  | **β** | **P** |  | **β** | **P** |
| Total score of ESS | 1.236e-02 | **0.000279** |  | -5.857e-03 | 0.244 |
| Possibilities of daytime sleepiness when sitting and reading | 5.877e-02 | **0.0155** |  | -1.802e-02 | 0.554 |
| Possibilities of daytime sleepiness when watching TV | 3.305e-02 | 0.130 |  | -2.871e-03 | 0.919 |
| Possibilities of daytime sleepiness when sitting inactive in public places | 6.492e-02 | **0.00448** |  | 4.331e-04 | 0.988 |
| Possibilities of daytime sleepiness when one as a passenger stays in a car for an hour without a break | 7.551e-02 | **0.00477** |  | -5.774e-02 | 0.0728 |
| Possibilities of daytime sleepiness when lying down to rest in the afternoon | -7.163e-03 | 0.796 |  | -3.392e-02 | 0.254 |
| Possibilities of daytime sleepiness when sitting and talking to someone | 6.852e-02 | **0.00436** |  | 8.495e-03 | 0.846 |
| Possibilities of daytime sleepiness when sitting quietly after lunch | 9.400e-02 | **0.000386** |  | -3.880e-02 | 0.233 |
| Possibilities of daytime sleepiness in a car, while stopped in traffic | 9.500e-02 | **1.05e-05** |  | -1.019e-05 | 1.000 |

MDS-UPDRS: Movement Disorder Society Unified Parkinson’s Disease Rating Scale;PD: Parkinson’s disease;HC: Healthy controls; ESS: Epworth sleep scale

| **Table 11. Analysis of indirect effect and direct effect between RBD and GDS in PD and HC individuals.** | | | | | | | | |
| --- | --- | --- | --- | --- | --- | --- | --- | --- |
| **Autonomic dysfunction assessments** | | **PD** | | |  | **HC** | | |
|  |  | **β** | **95%CI** | **P** |  | **β** | **95%CI** | **P** |
| SCOPA-AUT Total Score | IE | 0.0698 | 0.0354,0.11 | **<2e-16** |  | 0.0858 | 0.0163,0.18 | **<2e-16** |
|  | DE | 0.0610 | -0.0332,0.15 | 0.24 |  | -0.0647 | -0.2934,0.12 | 0.60 |
| SCOPA-AUT Cardiovascular Score | IE | 0.0558 | 0.0288,0.09 | **<2e-16** |  | 0.02000 | -0.00528,0.06 | 0.28 |
|  | DE | 0.0738 | -0.0141,0.16 | 0.08 |  | 0.00278 | -0.21339,0.19 | 0.92 |
| SCOPA-AUT Gastrointestinal (GI) Score | IE | 0.0509 | 0.0218,0.09 | **<2e-16** |  | 0.03460 | -0.00365,0.09 | 0.08 |
|  | DE | 0.0778 | -0.0138, 0.16 | 0.08 |  | -0.01160 | -0.22939,0.18 | 0.84 |
| SCOPA-AUT Pupillomotor Score | IE | 0.00806 | -0.00117,0.02 | 0.12 |  | 0.000504 | -0.013909, 0.03 | 0.84 |
|  | DE | 0.12042 | 0.03219,0.21 | **<2e-16** |  | 0.021310 | -0.180745,0.21 | 0.88 |
| SCOPA-AUT Sexual Dysfunction Score | IE | 0.00534 | -0.01296,0.02 | 0.60 |  | 0.02768 | -0.00698,0.08 | 0.28 |
|  | DE | 0.12435 | 0.03283,0.21 | **<2e-16** |  | -0.00493 | -0.22312,0.18 | 1.00 |
| SCOPA-AUT Thermoregulatory Score | IE | 0.03872 | 0.01415,0.07 | **<2e-16** |  | 0.01938 | -0.03780,0.09 | 0.64 |
|  | DE | 0.09021 | 0.00105,0.18 | 0.08 |  | 0.00241 | -0.22369, 0.19 | 0.84 |
| SCOPA-AUT Urinary Score | IE | 0.02231 | 0.00352,0.05 | **<2e-16** |  | 0.04450 | -0.00239,0.11 | 0.08 |
|  | DE | 0.10641 | 0.01736,0.19 | **0.04** |  | -0.02384 | -0.24839,0.17 | 0.72 |

RBD: Rapid eye movement sleep Behavior Disorder; GDS: Geriatric Depression Scale;PD: Parkinson’s disease;HC: Healthy controls; SCOPA-AUT:Scales for Outcomes in Parkinson’s disease- Autonomic; IE: Indirect Effect; DE:Direct Effect.

| **Table 12. Mediation of autonomic dysfunctions for the relationship between RBD and GDS in PD and HC individuals..** | | | | | | |
| --- | --- | --- | --- | --- | --- | --- |
| **Autonomic dysfunction assessments** | | **PD** | |  | **HC** | |
|  |  | **β** | **P** |  | **β** | **P** |
| SCOPA-AUT Total Score | a | 0.70242 | **4.35e-11** |  | 0.60933 | **1.43e-05** |
|  | b | 0.105923 | **5.61e-08** |  | 0.11814 | **0.0228** |
| SCOPA-AUT Cardiovascular Score | a | 0.0681596 | **1.17e-06** |  | 0.0297504 | 0.105 |
|  | b | 0.8524106 | **4.75e-09** |  | 0.57945 | 0.1559 |
| SCOPA-AUT Gastrointestinal (GI) Score | a | 0.198838 | **3.1e-08** |  | 0.085850 | **0.02298** |
|  | b | 0.2809798 | **9.11e-07** |  | 0.35994 | 0.0680 |
| SCOPA-AUT Pupillomotor Score | a | 0.019179 | 0.0804 |  | -0.023107 | 0.22132 |
|  | b | 0.415120 | **0.0311** |  | 0.03804 | 0.9241 |
| SCOPA-AUT Sexual Dysfunction Score | a | 0.10577 | **0.000353** |  | 0.134649 | **0.0176** |
|  | b | 0.0786095 | 0.2796 |  | 0.17881 | 0.1742 |
| SCOPA-AUT Thermoregulatory Score | a | 0.120860 | **2.94e-07** |  | 0.132645 | **0.000377** |
|  | b | 0.363045 | **3.28e-05** |  | 0.13010 | 0.5109 |
| SCOPA-AUT Urinary Score | a | 0.19634 | **0.000185** |  | 0.2595731 | **0.0016** |
|  | b | 0.127840 | **0.00132** |  | 0.14975 | 0.0981 |

RBD: Rapid eye movement sleep Behavior Disorder; GDS: Geriatric Depression Scale;PD: Parkinson’s disease;HC: Healthy controls; SCOPA-AUT:Scales for Outcomes in Parkinson’s disease- Autonomic; a: the direct effect of RBD on autonomic symptoms; b: the direct effect of autonomic symptoms on depression assessed by GDS.

| **Table 13. Analysis of indirect effect and direct effect between RBD and MDS-UPDRS Part I Depressed Mood in PD and HC individuals.** | | | | | | | | |
| --- | --- | --- | --- | --- | --- | --- | --- | --- |
| **Autonomic dysfunction assessments** | | **PD** | | |  | **HC** | | |
|  |  | **β** | **95%CI** | **P** |  | **β** | **95%CI** | **P** |
| SCOPA-AUT Total Score | IE | 0.01206 | 0.00605,0.02 | **<2e-16** |  | 0.00976 | 0.00102,0.02 | **0.04** |
|  | DE | 0.00955 | -0.01045,0.03 | 0.28 |  | 0.00725 | -0.02548,0.04 | 0.60 |
| SCOPA-AUT Cardiovascular Score | IE | 0.005397 | 0.000956,0.01 | **<2e-16** |  | 0.000989 | -0.005068,0.01 | 0.80 |
|  | DE | 0.015831 | -0.003517,0.03 | 0.08 |  | 0.019104 | -0.019806, 0.05 | 0.28 |
| SCOPA-AUT Gastrointestinal (GI) Score | IE | 0.00840 | 0.00324 ,0.02 | **<2e-16** |  | 0.01430 | 0.00430,0.03 | **<2e-16** |
|  | DE | 0.01298 | -0.00673, 0.03 | 0.20 |  | 0.00625 | -0.03189,0.04 | 0.72 |
| SCOPA-AUT Pupillomotor Score | IE | 0.002430 | 0.000134, 0.01 | **0.04** |  | 0.00015 | -0.00430, 0.00 | 0.84 |
|  | DE | 0.018810 | 0.000126 ,0.04 | 0.08 |  | 0.01983 | -0.01761, 0.05 | 0.32 |
| SCOPA-AUT Sexual Dysfunction Score | IE | 0.001659 | -0.001453,0.01 | 0.48 |  | 0.00849 | 0.00113,0.02 | **0.04** |
|  | DE | 0.019693 | 0.000466,0.04 | 0.08 |  | 0.01191 | -0.02683,0.05 | 0.52 |
| SCOPA-AUT Thermoregulatory Score | IE | 0.005054 | 0.000314,0.01 | **<2e-16** |  | 0.00339 | -0.00212,0.01 | 0.28 |
|  | DE | 0.016141 | -0.003118,0.03 | 0.08 |  | 0.01678 | -0.02229, 0.05 | 0.40 |
| SCOPA-AUT Urinary Score | IE | 0.004379 | 0.000895,0.01 | **<2e-16** |  | 0.0014 | -0.0058,0.01 | 0.72 |
|  | DE | 0.016845 | -0.001990,0.03 | 0.08 |  | 0.0154 | -0.0164,0.04 | 0.28 |

RBD: Rapid eye movement sleep Behavior Disorder; MDS-UPDRS: Movement Disorder Society Unified Parkinson’s Disease Rating Scale;PD: Parkinson’s disease;HC: Healthy controls; SCOPA-AUT:Scales for Outcomes in Parkinson’s disease- Autonomic;IE: Indirect Effect; DE:Direct Effect.

| **Table 14. Mediation of autonomic dysfunctions for the relationship between RBD and MDS-UPDRS Part I Depressed Mood in PD and HC individuals.** | | | | | | |
| --- | --- | --- | --- | --- | --- | --- |
| **Autonomic dysfunction assessments** | | **PD** | |  | **HC** | |
|  |  | **β** | **P** |  | **β** | **P** |
| SCOPA-AUT Total Score | a | 0.69427 | **1.03e-13** |  | 0.52233 | **2.28e-06** |
|  | b | 0.018298 | **9.45e-06** |  | 0.019100 | **0.0155** |
| SCOPA-AUT Cardiovascular Score | a | 0.0682670 | **4.9e-08** |  | 0.027146 | 0.0700 |
|  | b | 0.091921 | **0.00268** |  | 3.717e-02 | 0.6199 |
| SCOPA-AUT Gastrointestinal (GI) Score | a | 0.206615 | **1.01e-10** |  | 0.098165 | **0.0027** |
|  | b | 0.044761 | **0.000185** |  | 0.1413598 | **2.12e-05** |
| SCOPA-AUT Pupillomotor Score | a | 0.0231152 | **0.0262** |  | 0.0005253 | 0.974163 |
|  | b | 0.103667 | **0.00550** |  | -0.0310785 | 0.6550 |
| SCOPA-AUT Sexual Dysfunction Score | a | 0.089635 | **0.000328** |  | 0.115043 | **0.00628** |
|  | b | 0.021679 | 0.17056 |  | 7.167e-02 | **0.00637** |
| SCOPA-AUT Thermoregulatory Score | a | 0.1266529 | **1.99e-09** |  | 0.079983 | **0.0142** |
|  | b | 0.048525 | **0.00736** |  | 0.0442254 | 0.1964 |
| SCOPA-AUT Urinary Score | a | 0.18340 | **6.38e-05** |  | 0.20599 | **0.00244** |
|  | b | 0.025174 | **0.002770** |  | 0.0087305 | 0.507 |

RBD: Rapid eye movement sleep Behavior Disorder; MDS-UPDRS: Movement Disorder Society Unified Parkinson’s Disease Rating Scale;PD: Parkinson’s disease;HC: Healthy controls; SCOPA-AUT:Scales for Outcomes in Parkinson’s disease- Autonomic; a: the direct effect of RBD on autonomic symptoms; b: the direct effect of autonomic symptoms on depression assessed by MDS-UPDRS.
